# Physicochemical Characteristics, Antioxidant Properties, Aroma Profile, and Sensory Qualities of Value-Added Wheat Breads Fortified with Post-Distillation Solid Wastes of Aromatic Plants

**DOI:** 10.3390/foods12214007

**Published:** 2023-11-02

**Authors:** Chrysanthi Nouska, Maria Irakli, Miltiadis Georgiou, Anastasia E. Lytou, Adriana Skendi, Elisavet Bouloumpasi, Paschalina Chatzopoulou, Costas G. Biliaderis, Athina Lazaridou

**Affiliations:** 1 Laboratory of Food Chemistry and Biochemistry, Department of Food Science and Technology, School of Agriculture, Aristotle University of Thessaloniki, P.O. Box 235, 54124 Thessaloniki, Greece; cinouska@agro.auth.gr (C.N.); mgeorgiou@agro.auth.gr (M.G.); biliader@agro.auth.gr (C.G.B.); 2 Hellenic Agricultural Organization—DIMITRA, Institute of Plant Breeding and Genetic Resources, Thermi, 57001 Thessaloniki, Greece; alytou@aua.gr (A.E.L.); antrsken@teiemt.gr (A.S.); elisboul@abo.ihu.gr (E.B.); pchatzopoulou@elgo.gr (P.C.)

**Keywords:** bread texture, antioxidant activity, phenolic compounds, SPME-GC/MS, LC/MS, essential oil extraction by-products, oregano, rosemary, lemon balm, spearmint

## Abstract

The influence of incorporation of post-distillation solid wastes of the aromatic plants (SWAP), oregano, rosemary, lemon balm, and spearmint into wheat breads at 1% and 2% levels on their physicochemical and sensorial properties, and antioxidant and volatile profiles were investigated. SWAP breads had darker crumbs and crust and greener crumbs compared to the control, but rather similar loaf specific volume and textural attributes (crust puncture test and crumb Texture Profile Analysis). Although the mold growth on bread crumb surface was not inhibited by SWAP presence, LC-DAD-MS revealed a large increase in terpenoids, like carnosic acid (all SWAP), carnosol (rosemary) and carvacrol (oregano), phenolic (rosmarinic and salvianolic) acids and flavonoids in bread with SWAP inclusion, leading to enhanced antioxidant capacity (ABST, DPPH and FRAP assays). The distinct aromatic plant flavors were detected in the fortified breads by trained assessors and confirmed by SPME-GC/MS volatile analysis, showing high levels of terpenoids in SWAP breads, like carvacrol (oregano), caryophyllene (rosemary and lemon balm), and carvone (spearmint), and rendering the 2% fortification unacceptable by consumers. Nevertheless, breads with 1% oregano or rosemary waste had similar control overall acceptability scores, indicating that SWAP can be a promising ingredient for developing antioxidant-enriched wheat breads.

## 1. Introduction

Nowadays, consumer behavior is changing due to raised health concerns associated with food quality. In addition, healthy eating habits towards more nutritious foods are encouraged. Consuming foods rich in antioxidants or other bioactive compounds that can promote human health is highly recommended. Generally, the formulation of plain bread, blamed for its high glycemic index and being poor in antioxidants and bioactive compounds, is enlarging to match the need for better quality bread. Bread composition is thus changing by adopting various alternative ingredients to embrace this new trend [1,2,3,4]. The incorporation of flours from diverse raw materials rich in functional components, aiming to increase the content of fiber and bioactive compounds in breads, has increased during the last years, being visible by numerous published studies in the literature. The ultimate goal is to produce breads utilizing nutrient-dense ingredients (i.e., dietary fibers, phenolic antioxidants, n-3 fatty acids) to increase their functionality and health attributes [5]. A fast search in Scopus made to retrieve relevant literature containing keywords “bread AND dietary AND fiber” revealed 715 documents (641 articles, 52 reviews, 6 book chapters), whereas when using the search terms “bread AND antioxidant” gave 633 documents (584 articles, 35 reviews, 8 book chapters) (Last access 8 August 2023). These studies differ in the origin of raw materials, as well as the quantity and profile of the bioactive compounds present in the functional ingredient used for product fortification. Health-oriented research findings indicated that the properties of these breads produce acute changes in metabolism and physiology (e.g., appetite suppression, reduced diastolic blood pressure, improvements in glycemia, insulinemia, and satiety effect). However, Amoah et al. [6], in their review, have concluded that despite the encouraging positive results, the overall outcome is not enough for making a health claim since long trials are missing for studying the long-term health effects from the consumption of these products.

Phenolic and/or fiber-rich flours/extracts obtained from non-traditional sources (legumes, seeds, nuts, fruit, vegetables, tubers, herbs/weeds, leaves) are often used to formulate bakery products with the characteristics of a functional food [6]. Among the different sources of raw materials, medicinal and aromatic plants (MAPs) are traditionally accepted to provide a positive effect on human health due to the bioactive compounds they contain. Therefore, they are often recommended to be used as additives in the production of functional foods [1,3,7]. The studies on the incorporation of functional ingredients into bread are not limited to increasing the phenolic compounds content and/or the antioxidant activity but also deal with the evaluation of the effects these materials have on the physicochemical properties, sensorial attributes, and shelf life of bread.

Filipčev [8] summarized the literature that deals with the addition of different aromatic herbs to bread and bakery products and their effect on the product quality attributes. Multiple advantages of the incorporation of MAPs in a bread recipe have been reported. Besides the enrichment of bread with phytochemicals and the increase in its antioxidant capacity, positive results in terms of extended shelf life by providing a barrier against fungal growth have also been reported [9]. Nevertheless, several negative effects on the bread’s textural and sensorial attributes have been noticed. The addition of aromatic plants or spices/herbs was reported to weaken dough structure, reduce loaf specific volume, and affect bread sensorial acceptability [10]. This behavior was attributed to the increase in the fiber content that dilutes or disrupts the gluten network and the inhibition of yeast activity from the compounds with antimicrobial activity [11]. 

By-products originating from distillation of MAPs are recognized as being rich in phytochemicals with antioxidant capacity suitable for use as flours or for isolation of phytochemicals to be used in a multitude of applications [4,12,13,14,15]. Valorization of the agri-food industry by-products represents a strategy toward a more sustainable economy [4] that also offers new business opportunities [16]. In a study on the use of waste material from *Lavandula angustifolia* and *Melissa officinalis* distillation process to supplement wheat flour for bread production [17], the authors reported that the addition of only 5% residues tripled the amount of total dietary fiber, quadrupled the polyphenols and flavonoids and increased the shelf life against fungal or bacterial spoilage of the bread by 96 h. It seems that even post-distillation by-products from oregano and rosemary plants have antibacterial and antioxidant properties [18]. On the other hand, Vasileva et al. [17] observed that only a level of 2.5% lavender waste maintained the loaf specific volume, suggesting that the quality characteristics of bread are concentration-dependent. Properties given to the bread by adding MAPs depend not only on concentration but also on the type of plant material added. In a previous study, Skendi et al. [7] reported that thyme was the most potent antifungal against fungi compared to oregano and satureja, whereas Vasileva et al. [17] reported that lavender was more effective than melissa (lemon balm).

When designing these fortified breads, one must keep in mind that the different processes involved during breadmaking also affect the final amount of phytochemicals, texture, and sensorial parameters of bread. Breadmaking processes were reported to decrease the total phenolic content and antioxidant capacity of bread crumbs enriched with anthocyanin-rich plants [19]. Similarly, Skendi et al. [11] reported that the baking procedure decreased the level of phenolic compounds in fortified breads with oregano, thyme, and satureja, showing a decrease that could reach 22% of the theoretically expected values, depending on the aromatic plant material added. Despite the decrease due to the breadmaking process, a much higher phenolic content and antioxidant capacity are observed in breads supplemented with MAPs, compared to the control, a result that also depends on the fortification level [9].

The sensorial attributes of bread influence, to a great extent, the consumer’s acceptability; the color and aroma of bread are mostly affected by plant-originating flours influencing the final acceptance. Besides the studies employing sensory analysis with panelists, those characterizing the volatile profile of bakery products have been performed utilizing different head space GC-MS techniques [20]. Jensen et al. [21] tested α-tocopherol and rosemary extracts as antioxidative additives in bread to counteract the storage-related off-flavor development as a consequence of lipid oxidation. They reported that practically flavorless rosemary extracts did not have any effect on the aroma and flavor of fresh bread, as well as on the chemical stability of bread, whilst α-tocopherol resulted in a higher degree of rancid aroma and flavor [21]. On the other hand, Mildner-Szkudlarz et al. [22] report that phenolics added to a model bread significantly suppressed aroma generation during baking but inhibited acrylamide generation.

Being commonly consumed as a staple food, bread could be a practical way to deliver plant-based ingredients with positive health effects deriving from by-products of the essential oil industry of MAPs. Although some studies have focused on the effect of MAP addition on bread quality parameters, antioxidant profile, and sensorial attributes [11], very few studies are available on the influence of their waste by-products. Aromatic plants of the Lamiaceae family are rich in essential oils, comprising several species among the most traded globally. Oregano, rosemary, lemon balm, and spearmint include plant species belonging to the Lamiaceae family, with great potential to be used as natural antioxidants in the food industry. Skendi et al. [7] have summarized the literature concerning the abundance of phenolic compounds in solid wastes remaining after essential oil distillation of several aromatic plants, indicating that those of oregano, rosemary, lemon balm, and spearmint contained various groups of phenolic compounds in significant amounts. Thus, the present study aimed to investigate the effect of fortification of wheat flour with post-distillation solid wastes of four MAPs (oregano, rosemary, lemon balm, and spearmint) on wheat bread’s quality attributes (specific volume, color, texture, and sensory characteristics) as well as the profile of volatiles (aroma) and phenolic compounds, and the antioxidant capacity in order to explore the potential use of wheat bread as a carrier of such aromatic plant antioxidants, and to contribute to an understanding of how food wastes can be reduced and thereby their negative impact on environment.

## 2. Materials and Methods

### 2.1. Raw Materials

Wheat flour (type 70) with 13.62% moisture, 0.50% ash, 14.90% protein, 30.5% wet gluten, and 250 s falling number was gifted by Select Bakery SA (Sindos, Greece). The Greek oregano (*Origanum vulgare ssp. hirtum* L.), rosemary (*Rosmarinus officinalis* L.), lemon balm (*Melissa officinalis* L.), and spearmint (*Mentha spicata* L.) solid wastes were collected after steam distillation in a pilot-scale apparatus for approximately 2 h in order to remove the essential oils. The remaining solid wastes were oven-dried at 50 °C until reaching a moisture content of less than 10% and then ground in a laboratory mill (Retsch, Model ZM 1000, Haan, Germany) to pass through a 0.5 mm sieve. The dried powders of solid wastes remaining after distillation of the aromatic plants (SWAP) of oregano (ORE), rosemary (ROS), lemon balm (LEB), and spearmint (SPE) were kept at 4 °C before further treatments. 

### 2.2. Chemicals and Reagents

The analytical reagents folin-ciocalteu, 2,2-diphenyl-1-picryhydrazyl (DPPH), 2,2-azinobis-(3-ethylbenzthiazoline-6-sulphonic acid) (ABTS), and 2,4,6-tripyridyl-s-triazine (TPTZ) were purchased from Sigma-Aldrich (Steinheim, Germany). Analytical standards of gallic acid (GA), caffeic acid (CA), p-coumaric acid (pCA), trans-ferulic acid (FA), salicylic acid (SLA), and verbascoside (VER) were obtained from Sigma-Aldrich (Steinheim, Germany). Rosmarinic acid (RMA), eriodictyol (ERD), apigenin (API), catechin (CAT), carvacrol (CARV), and luteolin-7-O-glucoside (LUTGL) were purchased from Extrasynthese (Genay Cedex, France). Luteolin-7-O-rutinoside (LUTRU), naringenin (NAR), vicenin-2 (VIC), hesperisin (HESP), taxifolin (TAX), salvianolic acid B (SALB), carnosol (CARO), and carnosic acid (CARA) were obtained from Carbosynth (Berkshire, UK). All the other solvents used for the extraction of phenolic compounds, as well as the chromatographic analysis, were of HPLC or LC-MS grade.

### 2.3. Breadmaking Procedure

The dried powders of ORE, ROS, LEB, and SPE were used to fortify wheat breads at 1 and 2% levels of wheat flour substitution (Table 1). Water absorption values were determined by farinography (Brabender GmbH and CO, Duisburg, Germany) and were used to obtain the optimum level of added water into the dough formulations. The composition of all bread formulations is given in Table 1.

For the preparation of the composite breads, the wheat flour-SWAP mixtures were mixed with dry baker’s yeast, salt, and water in a professional spiral mixer (Resto Italia SK 10 MO, Urbino, Italy). After kneading for 30 min, the dough was left to rest for 20 min at room temperature, divided into 280 g pieces, shaped into round balls, rested for 10 min, formed into loaves, and finally placed into pans. Prior to baking at 180 °C for 28 min (air-o-stream combi oven, Electrolux Professional SpA, Pordenone, Italy), the samples were proofed for 30 min at 40 °C and 75% relative humidity. The breadmaking procedure was repeated three times for each bread formulation. Bread products were stored for up to 4 days at 25 °C in sealed polypropylene bags.

### 2.4. Physicochemical Parameters of Breads

A chromameter (Konica Minolta, CR-400 Series, Tokyo, Japan) was used to determine L*, a*, and b* parameters (CIE system) for evaluation of the crust and crumb color of the breads. The specific volume of the loaves was determined using a benchtop laser-based scanner (VolScan Profiler VSP600, Stable Micro Systems, Godalming, UK).

To assess the extent of staling of breads fortified with SWAP, the crumb and crust moisture content was determined in fresh (2 h after baking, 0 day) and stored breads for 4 days at 25 °C, according to AACC International 44-15.02 method [23]. Additionally, the Texture Analyzer TA.XT plus (Stable Micro Systems, Godalming, Surrey, UK) was used to evaluate bread texture for the same time intervals (at 0 day and 4 days of bread storage). To estimate crust hardness, a bread crust (40 mm length × 30 mm width × 5 mm height) was placed on the texture analyzer, and a penetration test was carried out using a 6.35 mm spherical stainless probe at 1 mm/s speed test [24]. For evaluation of the crumb texture, Texture Profile Analysis (TPA) was performed on a cylindrical crumb specimen (40 mm height × 30 mm diameter) using a 75 mm diameter platen probe (60% applied deformation; 5 mm/s test speed and 5 s delay time between the two compression cycles); crumb hardness, cohesiveness, and chewiness were estimated according to Armero and Collar [25]. For both crust and crumb tests, two specimens, at least, obtained from three different bread loaves for each breadmaking batch, were analyzed.

### 2.5. Surface Coverage of Breads by Mold during Storage

The shelf life of breads fortified with SWAP was also assessed using macroscopic observations of mold growth on the breadcrumb surface. Slices with 3 cm thickness were obtained from the middle of the bread loaf and stored in sealed polyethylene bags for 10 days at room temperature (25 °C). The degree of mold growth on the surface of the slices was quantified as a percentage of the bread area using image analysis with ImageJ 1.41o software. Three slices obtained from three different breads for each breadmaking batch were tested.

### 2.6. Preparation of Free and Bound Phenolic Extracts

An extraction procedure that allows to obtain the free and bound fractions of phenolics from bread samples was applied. This extraction procedure allows selective quantification of each fraction of phenolics, providing more information regarding the fate of phenolics and antioxidant activity in the final product. Free phenolic extracts of SWAP and freeze-dried fortified breads with SWAP were isolated as follows: SWAP (0.05 g) or bread samples (0.5 g) were mixed with 70% (*v*/*v*) aqueous methanol (20 mL or 5 mL, respectively) and extracted for 2 min by an ultrasonic homogenizer (Sonoplus model HD 4100, Berlin, Germany), consisting of a generator GM 4200, a converter UW 100 and a titanium probe TS 103 with 3 mm diameter and operating at 20 kHz under a working amplitude equal to 50%. The extracts were then centrifuged (Universal 320R, Hettich, Germany) at 4000 rpm (2680× *g*) for 10 min, and the supernatants were stored at −20 °C until further usage. Three replications were conducted for each sample.

The residual pellet of bread samples, obtained after removing free phenolic compounds, was further processed immediately for quantifying the bound phenolic extracts. Specifically, 20 mL of 4.0 M sodium hydroxide was added to the pellet, and the content was extracted on an ultrasonic bath (frequency 37 kHz, model FB 15051, Thermo Fisher Scientific Inc., Loughborough, UK) for 1.5 h at 40 °C. Then, the mixture was cooled, acidified to pH 2.0 with 37% hydrochloric acid, and centrifuged at 4000× *g* for 10 min. The acidic supernatant was transferred into a clean separatory funnel, extracted three times with 30 mL ethyl acetate for each time, then pooled together, and eventually dried under a rotary evaporator until dryness. The extracted residue was re-dissolved in 2 mL of 70% methanol in water (*v*/*v*), and the bound extracts were stored at −20 °C until further analysis. Three replications were conducted for each sample.

### 2.7. Spectroscopic Determination of Total Phenolics, Total Flavonoids, and Antioxidant Activity of Fortified Breads with SWAP 

#### 2.7.1. Determination of Total Phenolic Content (TPC)

The TPC of free and bound extracts was determined according to a method described previously [26]. The extracts (0.2 mL) were mixed with 0.8 mL diluted Folin–Ciocalteu reagent with water in a proportion of 1:10 (*v*/*v*). After 5 min, 2 mL of 7.5% sodium carbonate was added to the mixture, and the final volume of extracts was adjusted to 10 mL with distilled water. The absorbance was recorded at 725 nm after a reaction of 1 h in the dark at room temperature, and the TPC value was expressed as mg of gallic acid equivalents per g of SWAP sample (mg GAE/g) or 100 g of bread sample on a dry mass basis (mg GAE/100 g bread d.b.).

#### 2.7.2. Determination of Total Flavonoid Content (TFC) 

The TFC of free and bound extracts was determined according to a colorimetric assay with aluminum chloride, as described by Bao et al. [27]. The extracts (0.3 mL) were mixed with 0.225 mL of 5% sodium nitrite (*v*/*v*), followed by the addition of 0.225 mL of 10% aluminum chloride hexahydrate (*v*/*v*) and 0.750 mL of NaOH (2 N). After 20 min of reaction, the absorption was recorded at 510 nm, and the TFC values were expressed as mg of catechin equivalents per g of SWAP samples (mg CATE/g) or 100 g of bread sample on a dry mass basis (mg CATE/100 g bread d.b.).

#### 2.7.3. ABTS Radical Scavenging Assay 

The ABTS assay was carried out according to the protocol of Re et al. [28]. The standard ABTS solution was diluted with water by adjusting the absorbance of the solution to 0.7 ± 0.02 at 734 nm. Then, 100 μL free or bound phenolic extract was mixed with 3.9 mL ABTS working solution, vortexed for 1 min, and then stored in the dark. After 5 min, the absorbance was recorded at 734 nm against a blank. The calibration curve was performed by plotting the respective absorbance values at different Trolox concentrations vs. % ABTS scavenging activity. The ABTS values were expressed as mg Trolox equivalents per g for the SWAP sample (mg TE/g) or 100 g of bread sample on a dry mass basis (mg TE/100 g bread d.b.).

#### 2.7.4. DPPH Radical Scavenging Assay 

The DPPH assay was conducted according to a previous report [29] with slight modifications. Briefly, 100 μL of each extract was mixed with 2.85 mL of freshly prepared 0.1 mM DPPH in methanol, and the decrease in absorbance was measured at 516 nm after 5 min of reaction; the calibration curve was performed by plotting the Trolox concentration and % DPPH scavenging activity. The DPPH values were expressed as mg Trolox equivalents per g for the SWAP sample (mg TE/g) or 100 g of bread sample on a dry mass basis (mg TE/100 g bread d.b.).

#### 2.7.5. Ferric Reducing Antioxidant Power (FRAP) Assay 

The FRAP assay was carried out according to the method of Benzie and Strain [30]. Briefly, fresh FRAP reagent was prepared by mixing 20 mM ferric chloride solution, 10 mM TPTZ (2,4,6-tripyridyl-s-triazine) solution in 40 mM HCl, and 0.3 mM acetate buffer pH 3.6 in a proportion of 1:1:10, respectively. 100 μL of appropriately diluted extracts were incubated with 3 mL FRAP working solution at 37 °C, and exactly after 4 min, the absorbance was recorded at 593 nm. Trolox was used as an antioxidant standard, and the FRAP values were also expressed as mg Trolox equivalents per g for the SWAP sample (mg TE/g) or 100 g of bread sample on a dry mass basis (mg TE/100 g bread d.b.).

### 2.8. HPLC-DAD-MS Analysis of Phenolic Compounds

A Shimadzu Nexera HPLC-DAD-ESI-MS system (Kyoto, Japan) operating using Lab Solutions LC-MS software (version 5.97.1, Shimadzu, Kyoto, Japan) was used for the identification and quantification of the phenolic compounds [11]. The free and bound phenolic extracts were filtered through a 0.22 μm PTFE syringe filter and, after the appropriate dilution, were put into the autosampler with an injection volume of 10 μL. The separation was performed in a Poroshell 120 EC-C_18_ analytical column (4.6 × 150 mm, 4 µm) (Agilent Technologies, Inc., Santa Clara, CA, USA) with a flow rate of 0.5 mL/min. The mobile phase A was water containing 0.1% formic acid, and B was acetonitrile: 0 min, 15% B; 3 min, 25% B; 12 min, 35% B; 24 min, 65% B; 24.5 min, 100% B; 30 min, 15%, and kept at 15% B for another 15 min. For MS analysis, an electrospray interface was used with +4.5 kV and 20 V interface and curved desolvation line (CDL) voltages, respectively. The mass spectrometer was operated using negative ionization mode with a full scan *m*/*z* 100–1000.

Phenolic compounds were tentatively identified in comparison with the retention profile, UV–Vis spectra, and mass spectra of unknown peaks with those of authentic standards or with literature data. The majority of phenolic compounds were quantified by selecting ion monitoring mode (SIM) of standard solutions, where the quantification calibration curves of the standard were created using an internal standard method using salicylic acid (1 µg/mL), while the quantification calibration curves of carvacrol created at a monitoring wavelength of 280 nm using the DAD system. When standards were unavailable, compounds with similar structures were used instead to perform quantification of the phenolic compounds. Specifically, lithospermic acid A, as well as other salvianolic acid isomers, were quantified as salvianolic acid B equivalents. 

### 2.9. Aroma Profile Analysis Using SPME-GC/MS

A solid-phase microextraction (SPME) fiber (DVB/CAR/PDMS) was used to extract volatile constituents from homogenized SWAP samples and the fortified breads. SPME was performed in 20 mL vials, sealed with PTFE-Silicone septa, containing 0.1 g of the homogenized sample (SWAP) or 1.0 g (bread). After 15 min of heating in a water bath at 50 °C, the fiber was introduced into the vial for an additional 30 min in order to capture the volatiles from the headspace.

Gas Chromatography–Mass Spectrometry (GC/MS) analysis was carried out using a Shimadzu GCMS QP-2010 Ultra system operated with the accompanying GCMS Solution software (version 4.52). The volatile components adsorbed on the SPME fiber were desorbed in the injection inlet at 240 °C (split ratio 1:10) for 5 min and separated on a GC column DB-5MS (30 m × 0.25 mm, 0.25 μm) obtained from Agilent Technologies (Santa Clara, CA, USA). Helium was employed as the carrier gas at a constant linear velocity of 36 cm/s. The initial oven temperature was set at 40 °C for 5 min, then it was raised at 5 °C/min to 180 °C, at 30 °C/min to 240 °C, and held there for 5 min. The temperature of the ion source and interface was set at 230 °C and 240 °C, respectively.

For identification, a homologous series of n-alkanes (C7–C22) was analyzed to calculate the Kovats index of each compound and compared with those reported in the literature. Additionally, the identification was achieved by matching their mass-spectra with those of mass-spectra libraries (NIST 98, Willey). The relative content of each compound was calculated as a percent of the total chromatographic area, and the results are expressed as means of three replicates.

### 2.10. Sensory Analysis

To evaluate the acceptability of fortified breads with SWAP, sensory analysis by an untrained panel was performed; 75 untrained assessors used a hedonic scale ranging from 1 (highly unpleasant) to 9 (highly enjoyable) to rate the overall acceptability of the bread samples. The first results indicated that fortified breads with 2% level of wheat flour substitution were not acceptable (mean values < 4) due to the intense flavor of the respective plant solid residue, which was described as unpleasant and bitter. Therefore, only breads with added 1% SWAP as a substitution for wheat flour were subjected to further sensory analysis. A group of 15 trained assessors, who consumed bread at least once a week, were recruited to evaluate the samples using a 9-point hedonic scale, where 1 represented a low intensity, and 9 represented a high intensity for each of the sensory attributes tested. The evaluated parameters included crumb and crust color, typical wheat and non-wheat aroma and taste, bitter taste, and the respective ORE, ROS, LEB, or SPE aroma and taste for each tested SWAP material.

### 2.11. Statistical Data Analysis

Mean ± standard deviations of all evaluated parameters were calculated from measurements of the three breadmaking batches. Comparison of means was performed using one-way analysis of variance (ANOVA) according to Tukey’s test (IBM SPSS statistical software, version 22.0, IBM Corp., Armonk, NY, USA). The two-way ANOVA was also used to examine the overall effects of the two factors examined: type of SWAP and level of wheat flour substitution. Possible associations among the phenolics and antioxidant capacity of the bread were estimated by Pearson’s correlation. Statistical significance was defined at *p* < 0.05.

## 3. Results and Discussion

### 3.1. Physical Characteristics of Fortified Wheat Breads with SWAP

**Figure 1:**
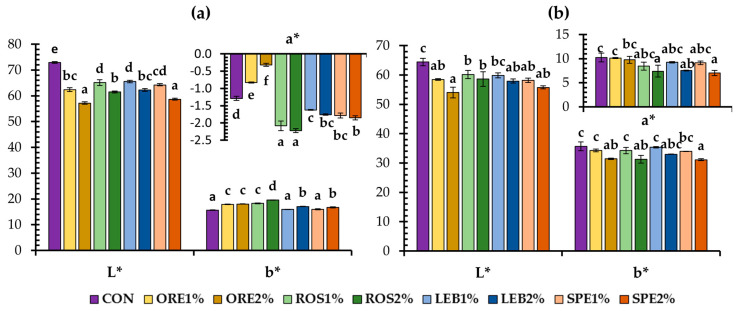
Color parameters of crumb (**a**) and crust (**b**) of wheat breads fortified with 1 and 2% oregano (ORE), rosemary (ROS), lemon balm (LEB), and spearmint (SPE) post-distillation solid waste residues; mean values with a different letter for the same parameter are significantly different (Tukey’s test, *p* < 0.05).

The color of the crust and crumb of the wheat bread was influenced by the inclusion of SWAP materials (Figure 1). A darker crust and crumb color (lower L* values) were observed for all SWAP-supplemented breads, compared to those of control. A similar trend was also reported in previous studies on breads fortified with aromatic plants [9,31], which was attributed to the light brown color of the used aromatic plant powders [31]. In most cases, breads supplemented with 2% of SWAP showed reduced crust redness and yellowness (lower a* and b* values, respectively), as shown in Figure 1b. On the other hand, a crumb of breads with LEB2% and SPE2% exhibited higher b* values compared to control bread, while ORE and ROS wastes resulted in increased yellowness at both 1 and 2% supplementation levels (Figure 1a). Additionally, the crumb a* values did not show a clear pattern, as the inclusion of ROS, LEB, and SPE solid wastes at both 1 and 2% levels resulted in a more greenish crumb, compared to the control, whereas only the breads fortified with the ORE solid residues exhibited less intensity of the green hue than the control; the highest crumb green hue was observed for the ROS waste residues containing breads. 

**Figure 2:**
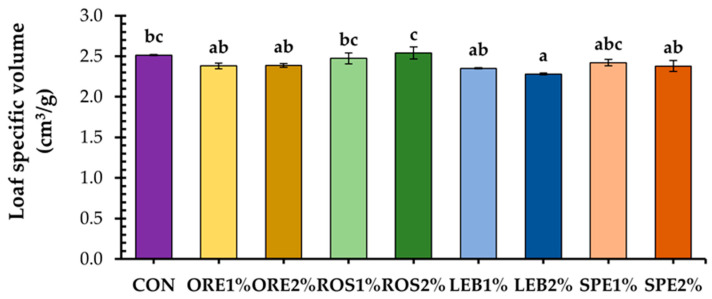
Loaf specific volume of wheat breads fortified with 1 and 2% oregano (ORE), rosemary (ROS), lemon balm (LEB), and spearmint (SPE) post-distillation solid waste residues; mean values with a different letter are significantly different (Tukey’s test, *p* < 0.05).

Loaf specific volume was affected slightly only by the inclusion of LEB2% in the fortified breads (Figure 2). This could be attributed to the relatively low level of wheat substitution by SWAP as it is well known that the substitution of wheat flour with other components can result in a decrease in the specific volume of the loaf due to interference or/and dilution of the gluten network [32]. Thus, Vasileva et al. [17] have noted that the incorporation of lavender waste only at 5% concentration and melissa waste at both 2.5 and 5.0% levels into wheat bread formulations resulted in decreased loaf specific volume, indicating that this quality attribute can be depended on the concentration of the added by-product and the plant species from which is originated.

### 3.2. Shelf-Life of Wheat Breads Fortified with SWAP 

#### 3.2.1. Changes in Bread Texture

**Figure 3:**
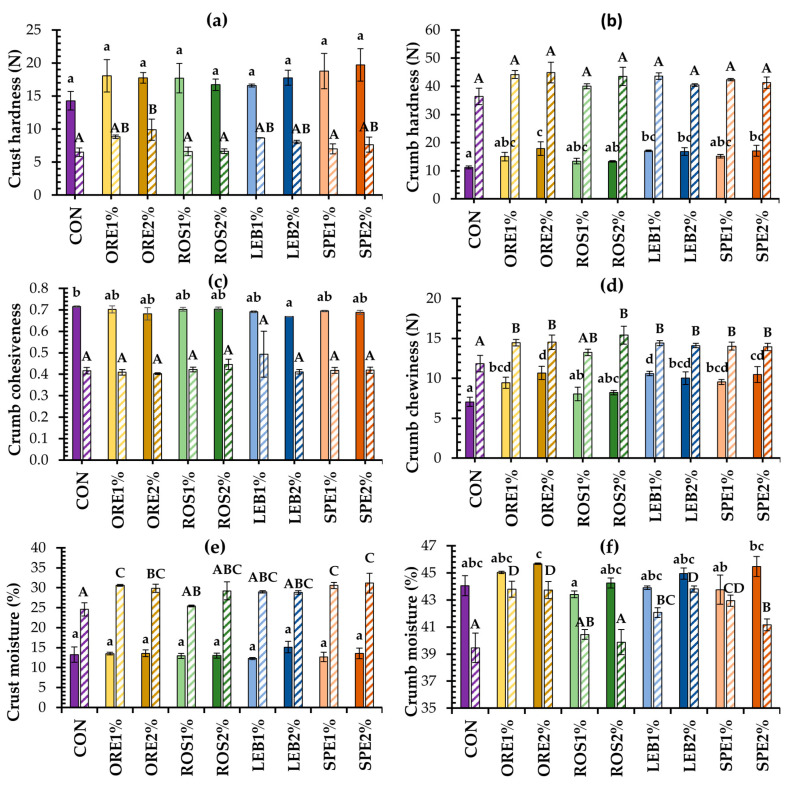
Textural properties (**a**–**d**) and moisture content (**e**,**f**) of wheat breads fortified with 1 and 2% oregano (ORE), rosemary (ROS), lemon balm (LEB) and spearmint (SPE) post-distillation solid waste residues stored for 0 (solid columns) and 4 (hatched columns) days at 25 °C; mean values for the same parameter with a different lowercase letter for the fresh (0 day) breads and different uppercase letters for the stored (4 days) breads are significantly different (Tukey’s test, *p* < 0.05).

The staling process of control and SWAP-enriched breads was evaluated by recording the changes in crust and crumb texture characteristics using the puncture and TPA tests, respectively, as well as the changes in their moisture contents between fresh (0 day) and stored (4 days × 25 °C) products (Figure 3a–f). As expected, for all bread formulations, storage resulted in decreased crust hardness (Figure 3a) and crumb cohesiveness (Figure 3c), along with increasing values of crumb hardness (Figure 3b) and chewiness (Figure 3d), all associated with bread staling events. These changes are attributed to the transfer of water from the crumb to the crust, as shown by the increase in crust moisture content (Figure 3e) and the decrease in crumb moisture (Figure 3f) upon storage for all tested breads. However, stalling process can also be promoted by the re-distribution of water at the molecular level among the various ingredients present in the bread matrix, leading to gluten transition from a rubbery to a glassy state as well as retrogradation of the amylopectin molecules (reordering of starch chains) that can also contribute to a more compact and stiff gluten-starch composite network and thus, to a harder bread crumb structure [32,33,34,35].

In most cases, the presence of plant solid waste residues in both fresh and stored breads did not have an impact on their crust hardness (Figure 3a) and crumb cohesiveness (Figure 3c), which are indices of crust crispness and crumb friability, respectively. On the other hand, the inclusion of oregano and spearmint waste at 2% level and lemon balm at both supplementation levels resulted in harder crumb. Compared to control, this adverse effect was eliminated during storage, resulting in similar crumb hardness for all tested breads after 4 days of storage (Figure 3b). Additionally, the crumb chewiness of the fresh fortified breads was significantly higher than that of control, except for the rosemary waste-containing bread at both fortification levels, while at the end of the storage period, all SWAP-containing breads, except ROS1%, exhibited higher crumb chewing values than control product (Figure 3d).

Variability in crumb textural attributes among the tested samples can be ascribed to differences in the water re-distribution and the accompanying phase transition events occurring upon staling in both gluten and starch at a molecular level. Wastes of aromatic plants are rich in dietary fibers, such as cellulose, hemicellulose, and lignin [14,17], which could increase the water-holding capacity of bread or compete for water with the starch component in a composite bread matrix and, thus, decrease starch retrogradation; both these processes contribute to a softer crumb. On the other hand, fibers can also compete with wheat proteins for water or interrupt or/and dilute the gluten network and, therefore, reduce its water retention capacity, leading the gluten to a glassy state and, finally, to crumb hardening [35,36]. Crust and crumb moisture contents of all SWAP breads did not significantly differ in the freshly made products (Figure 3e,f). However, after 4 days of product storage, the crust of ORE- and SPE-containing breads, as well as the crumb of all SWAP-breads, except ROS-containing samples, displayed higher moisture contents than the respective control; this is probably due to the presence of dietary fibers in the plant waste materials and their high capacity for water absorption.

#### 3.2.2. Mold Growth in Breads

**Figure 4:**
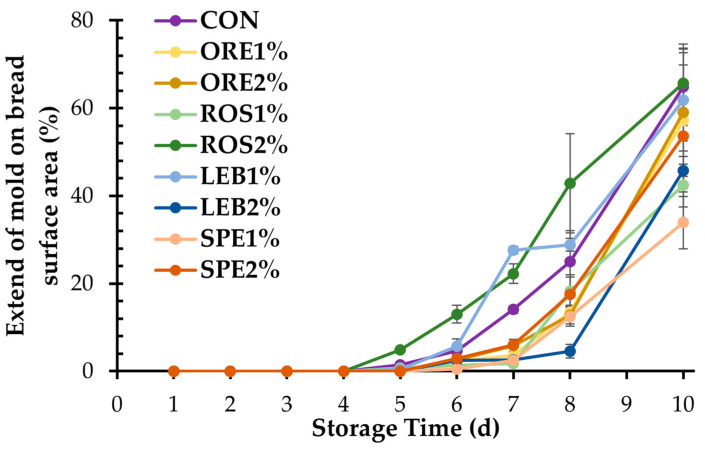
Surface coverage by mold in the crumb of wheat breads fortified with 1 and 2% oregano (ORE), rosemary (ROS), lemon balm (LEB), and spearmint (SPE) post-distillation solid waste residues during 10 days of storage at 25 °C.

**Figure 5:**
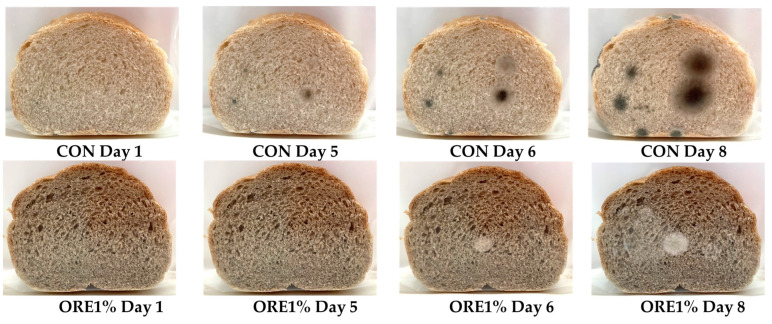
Representative images depicting the extent of mold coverage on the crumb surface of wheat bread without (control) and with the inclusion of 1% oregano (ORE) post-distillation solid waste residues in the bread formulation during storage at 25 °C.

Mold surface coverage in breads during storage at 25 °C is presented in Figure 4 and Figure 5. The appearance of mold growth on CON, ROS1%, ROS2%, and LEB1% was observed after 5 days of storage, while all other samples showed visible mold growth on the 6th day of storage. Although SWAP is rich in phenolic compounds, which could potentially extend the shelf life of the fortified breads by eliminating mold growth, their incorporation into wheat breads at low levels of up to 2% did not prove to be sufficient for preventing mold spoilage and increasing the storage stability of the fortified breads since mold spoilage was only retarded by one day, compared to control product. Additionally, higher moisture contents in the crumbs found on the 4th day of storage for most of the SWAP breads (Figure 3f), compared to control, which can favor mold growth, might overshadow the potential anti-fungal activity of the aromatic plant components. 

Similar findings were also obtained when lemon balm solid waste at 2.5 and 5.0% levels were included in wheat bread formulations, showing the same results as a control product or extending the bread’s shelf life by one day. On the other hand, the inclusion of lavender up to 10% wheat flour substitution resulted in delayed fungal spoilage, with mold appearing three days later than in control breads [17]. Delayed fungal growth has also been observed by other authors for breads fortified with *Cyperus rotundus rhizome* [37] and hop [38] extracts. Therefore, it can be concluded that mold spoilage can be inhibited depending on the type and the level of MAPs incorporated into the bread formulations.

### 3.3. TPC, TFC, and Antioxidant Activity of SWAP and Fortified Wheat Breads

**Figure 6:**
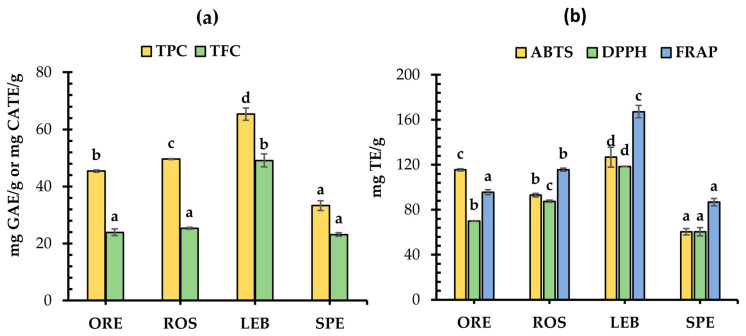
Total phenolic (TPC) and total flavonoid (TFC) contents (**a**) and antioxidant activity evaluated using ABTS, DPPH, and FRAP assays (**b**) of the post-distillation solid waste residues of oregano (ORE), rosemary (ROS), lemon balm (LEB) and spearmint (SPE); GAE: gallic acid equivalents were used for expressing TPC content; CATE: chatechin equivalents were used for expressing TFC content; TE: Trolox equivalents were used for expressing antioxidant capacity, as evaluated by the ABTS, DPPH and FRAP assays; mean values for the same parameter with different letter are significantly different (Tukey’s test, *p* < 0.05).

The TPC and TFC of free phenolic extracts of ORE, ROS, LEB, and SPE post-distillation solid wastes varied from 33.30 to 65.36 mg GAE/g and 23.17 to 49.13 mg CATE/g, respectively (Figure 6a), with LEB exhibiting the highest TPC and TFC values. Regarding TFC, the rest of the post-distillation solid wastes did not differ significantly (*p* > 0.05) among them, whereas the TPC values decreased in the following order: LEB > ROS > ORE > SPE. This ranking remained the same for the DPPH values, while the ABTS and FRAP values showed a slightly different trend; among the post-distillation waste residues tested, with all three used assays, the LEB sample showed the highest antioxidant capacity (Figure 6b). The levels of TPC and TFC values significantly correlated with the antioxidant capacity as evaluated using the ABTS, DPPH, and FRAP assays, as evidenced by the high correlation coefficients of 0.840, 0.971, 0.957, and 0.641, 0.924, 0.962, respectively (*p* < 0.01). These coefficients reveal the high degree of association of phenolic and flavonoid content with the antioxidant capacity and the potential that these materials could have in improving the antioxidant status of food products fortified with these residues. 

In general, solid wastes obtained from the distillation of MAPs of the Lamiaceae family are reported to have high TPC, TFC, and antioxidant activity [11], with lemon balm exhibiting similar values to that of spearmint but higher than oregano, sage, rosemary, and satureja. In their study, Alice et al. [39] reported that by-products from the hydro-distillation of *Origanum vulgare* along with *Melissa officinalis* showed the highest TPC compared to the other plants of the Lamiaceae family (*Salvia officinalis* L., *Mentha piperita* L., *Thymus vulgaris* L., and *Hyssopus officinalis* L.). Similarly, Ulewicz-Magulska and Wesolowski [40] have reported that oregano and thyme showed the highest contents of phenolics followed by melissa and rosemary. Differences in the amount of phenolics and the antioxidant capacity found could be not only due to the plant type, but also the result of different distillation, drying and extraction methods that can affect the remaining phenolics in the plant residues.

It is obvious that breads supplemented with SWAP material had significantly higher free and total TPC, TFC, and antioxidant capacity than the control bread (Table 2). Thus, it seemed that these functional breads fortified with SWAP had improved antioxidant properties, especially in the formulations made with a higher supplementation level (2%). In general, the bread supplemented with LEB residue contained the highest amount of TPC, TFC, and antioxidant capacity among all tested breads, which is in agreement with the findings for the respective parameters of the SWAP raw materials of breads (Figure 6), whereas the ORE breads exhibited the lowest values (Table 2). Additionally, it seemed that free TPC in bread samples represented 22.1 to 49.0% of the total TPC, while free TFC corresponded to 38.4 to 77.5% of the total TFC, suggesting that most of the TPC in breads are present in a bound form. 

In general, when considering both phenolic fractions, free and total, the antioxidant capacity of the samples, regardless of the assay applied (ABTS, DPPH, FRAP), can be almost entirely attributed to the TPC and TFC content since very high positive correlation (greater than 0.866, *p* < 0.01) were found among these parameters. The results of the two-way ANOVA (Table 2) revealed the significance of the independent variables (plant waste and level) and their interaction (plant waste × level) on the dependent variables (TPC, TFC, ABTS, DPPH, and FRAP) of the breads. It seemed that the type of plant waste, the level of supplementation, and their interactions affected the amount of total TPC, TFC, ABTS, DPPH, and FRAP. On the contrary, there was no significant effect of waste plant species on mean values of free TPC (*p* = 0.090) and SWAP supplementation level (1% and 2%) on the antioxidant capacity of free phenolic compounds present in breads when estimated using FRAP assay (*p* = 0.059). In addition, it seems that the interaction factor (plant waste × level) has no effect (*p* > 0.05) on free TPC, TFC, and their antioxidant capacity, as evaluated using all three ABTS, DPPH, and FRAP methods.

Generally, breadmaking negatively affected mostly the free fraction of phenolics, flavonoids, and their antioxidant capacity. The breadmaking process decreased the free TPC, TFC, and antioxidant capacity of the supplemented bread samples, except for the TPC and TFC of bread with SPE that showed an increase (TPC: 15.1 and 13.6%, TFC: 4.4 and 32.5%, for 1 and 2% supplementation level, respectively) compared to the theoretically expected values (Table 2). The degree of loss of these antioxidants during baking depended on the type of waste plant material used to fortify the breads. A maximal decrease from the expected theoretical values of 20.2 and 42.4% was observed for TPC and TFC values, respectively. However, a higher than 50% decrease in the antioxidant capacity (ABTS, DPPH, FRAP), compared to the expected theoretical values, was also noted, suggesting that phenolic compounds with the highest antioxidant capacity are those being more thermolabile. 

Similarly, Peng et al. [41] reported that thermal processing caused around a 30–40% decrease in the antioxidant activity of bread supplemented with grape seed extract. On the contrary, Abdel-Aal and Rabalski [42] reported that the baking process improved DPPH and ABTS antioxidant activities in bread despite the reduction observed after dough preparation. In addition, Yu and Beta [43] reported that the free phenolic content of purple wheat varieties significantly (*p* < 0.05) increased during mixing, fermenting, and baking (65% to 68%). This was most likely due to the complex phenomena occurring during baking, which involve starch gelatinization/pasting, protein denaturation, and Maillard reactions [44] as well as possible decomposition of some polymeric phenolic compounds to simpler molecules during baking. The differences reported in the literature for these can be a combination of different factors (i.e., type of plant material, quantity added, baking procedure).

### 3.4. Phenolic Profiles of SWAP Materials and Fortified Wheat Breads

Besides the determination of TPC and TFC with spectroscopic methods, the major phenolic compounds in SWAP (ORE, ROS, LEB, and SPE) extracts and fortified breads with SWAP were identified using LC/DAD/MS comparing their retention time, *m*/*z*, and UV spectra with standards or/and literature data and quantified (Table 3). It is obvious that the main group of phenolic compounds in LEB- and SPE-free extracts were the phenolic acids (PAs > 90% of the total quantified phenolics), while in ROS extract, phenolic terpenoids were the dominant phenolic group (PTs > 91% of the total quantified phenolics). On the other hand, in ORE-free extract, these two phenolic groups (phenolic acids and phenolic terpenoids) were present at similar levels. Regarding individual phenolics, rosmarinic acid (RMA) is undoubtedly the major phenolic acid for all four SWAP-studied extracts, followed by salvianolic acid isomers (SALA, SALB, and SALI) and caffeic acid (CA). Carnosic acid (CARA) was the only phenolic terpenoid present in all four SWAP extracts, while carvacrol (CARV) was detected only in oregano waste extract and carnosol (CARO) in rosemary extract. Total flavonoids were found in small but similar amounts in LEB and ROS extracts, while the ORE and SPE extracts have much higher levels. Vicenin-2 (VIC) and luteolin-7-O-glucoside (LUTGL) were present in all SWAP extracts but at less than 1%, except for the ORE extract. The amount of total quantified phenolics decreased in the following order: ROS > LEB > ORE > SPE. It seemed that TPC determined spectroscopically followed a slightly different order: LEB > ROS > ORE > SPE (Figure 6a).

In the case of bread samples, alkaline hydrolysis was applied in order to isolate bound phenolic compounds, as phenolic compounds form complexes with proteins via hydrogen, covalent, or ionic bonds and hydrophobic interactions [45]. Figure S1 (supplementary data) presents the chromatograms of soluble-free extracts of fortified wheat breads with ORE, ROS, LEB, and SPE post-distillation solid waste residues at 2% substitution level as well as the soluble-free and insoluble-bound (inset, Figure S1) phenolic extracts of control bread. Besides the main phenolics present in the SWAP-free extracts (Table 3), three other phenolic acids, trans- and cis-ferulic acids (t-FA and c-FA, respectively) and p-coumaric acid (pCA), were detected in both free and bound bread extracts (Figure S1 and Table 4). Sosulski et al. [46] reported that FA and p-CA exist in the form of free acids, soluble esters, and insoluble esters in cereal grains, and therefore, these two phenolic acids and caffeic acid (CA) were the only detected phenolic compounds in the control bread (Figure S1 and Table 4).

The main phenolics found in the control bread and supplemented breads with 1 and 2% SWAP are summarized in Table 4. It is evident that supplemented breads, regardless of the amount of SWAP added, have significantly higher levels of total quantified phenolics than control. Moreover, the higher the supplementation level, the higher the total phenolic compounds content; however, the baking process decreased the free total phenolics in the case of breads fortified with ORE and ROS post-distillation solid wastes (25 and 40%, respectively), compared to the theoretically expected values. On the other hand, an increase was observed in the case of breads fortified with LEB and SPE solid wastes (5 and 34%, respectively). In terms of the breadmaking process, baking decreased the content of all studied phenolic acids except for CA. In their study, Rahman et al. [47] reported that heat treatments quantitatively increased pCA, CA, and FA in brewers’ spent grain. On the contrary, Maghsoudlou et al. [48] reported that the roasting process adversely affects the amount of pCA in quince fruit. In another study, it was reported that autoclaving of oat grains increased pCA and FA while drastically decreasing CA [49]. These authors noticed a variation in the amount of phenolics depending on the food matrix and the process applied. Factors such as the food matrix, type of phenolics, and location, as well as heat treatment duration and intensity, influence the final phenolic content of thermally treated plant materials [50]. 

### 3.5. Aroma Profile of Fortified Wheat Breads with SWAP

The volatile compounds found in SWAP solid wastes, as well as the aroma profile of the control and SWAP-fortified breads, are presented in Table 5. In the control bread sample, the most abundant volatile compound that is responsible for the characteristic aroma of bread was the 1-butanol-3-methyl. Among other alcohols, 1-butanol-2-methyl, hexanol, and phenethyl alcohol were also important for bread aroma, derived from baker’s yeast alcoholic fermentation, providing fruity, malty, and floral aroma notes to wheat bread [51,52,53,54]. Aldehydes such as hexanal, heptanal, and benzaldehyde, derived from the oxidation of fatty acids and microorganic metabolism during fermentation, usually are linked to grass, rancid, and fatty off-flavors, but also to fruity, almond, and sweet pleasant flavor notes; these volatile compounds are often detected in wheat and gluten-free breads and largely contribute to their aroma [51,52,53,54]. 

With regard to SWAP samples, β-caryophyllene was the most abundant compound in ROS and LEB solid wastes, reaching 37.5 and 39.0% of the total volatile compounds, respectively, followed by 1,8-cineole (eucalyptol), bornyl acetate, and α-terpineol in ROS and germacrene D, and δ-cadinene in LEB (Table 5). A significant decrease in the typical aroma compounds of ROS and especially of 1,8-cineole in SWAP wastes and mainly in the fortified breads (Table 5) (1,8-cineole has a eucalyptus-like, camphoraceous odor [55]) can result in products that can be exploited by the bakery sector; i.e., the SWAP can provide high levels in phenolic antioxidants, without drastically affecting the sensory characteristics of the end product. Both 1,8-cineole and camphor (the latter has not been found in the ROS waste in the present study) are the predominant volatile compounds in rosemary essential oil [55]. Although 1,8-cineole on its own has a pleasant scent, it should be considered that at high concentrations, it may not fit well on breads. In LEB solid waste, compounds such as citronellal, neral, and geranial, which are characteristic volatile compounds of the LEB plant and its essential oil [56], were detected in significantly lesser amounts, while were absent in fortified breads (Table 5). 

Compounds such as carvacrol in ORE and carvone in SPE were maintained in both the solid waste residues and the respective fortified breads at high levels, thus largely contributing in the total volatile compounds, >40% (Table 5) and providing to the fortified bread products the intense oregano and spearmint odor, respectively [57,58]. It is worth noting that thymoquinone was detected in high concentrations in fortified ORE breads, possibly due to the transformation of carvacrol to thymoquinone, as a result of the selective hydroxylation of the aromatic ring followed by its subsequent oxidation during baking [59]. Additionally, β-caryophyllene which is linked to a pleasant spicy-earthy note [60], was the predominant volatile for ROS and LEB wastes and was largely preserved in the ROS-containing breads, though it was significantly decreased in LEB-fortified breads (Table 5).

### 3.6. Sensory Analysis of Fortified Wheat Breads with SWAP

**Figure 7:**
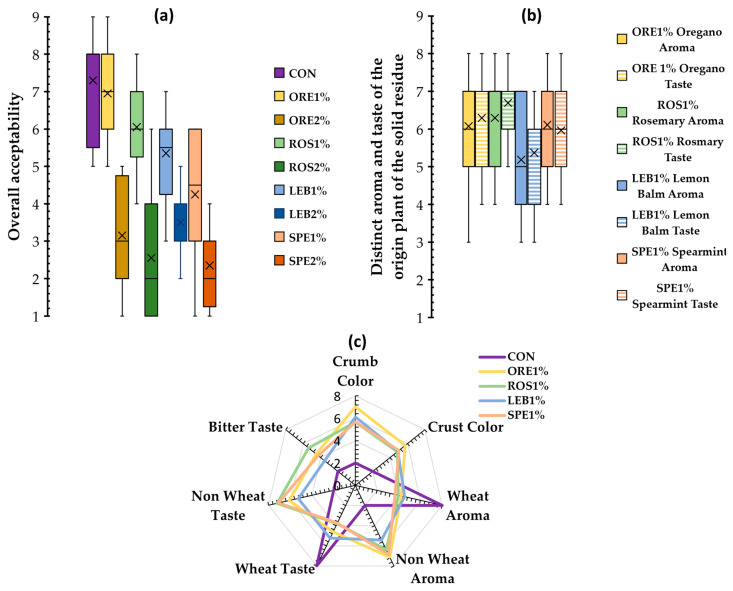
Overall acceptability (**a**), distinct aroma and taste of the plants from which originated the post-distillation solid waste residues (**b**) and sensory attributes (**c**) of wheat breads fortified with 1 and 2% oregano (ORE), rosemary (ROS), lemon balm (LEB) and spearmint (SPE) post-distillation solid waste residues; in the Boxplot charts, the interquartile range of values, from 25th to 75th per-centile, is represented by the “Boxes”. Median values are symbolized by ‘-’, mean values are symbolized by ‘x’, whereas the highest value is shown by the ‘upper bars’ and the lowest value is shown by the ‘lowest bars’.

The overall acceptability scores (Figure 7a) indicated that the ORE1% sample was the most acceptable product among the tested fortified breads followed by ROS1%. These samples also presented relatively low variability and received high mean scores of overall acceptability, higher than 6.0 and close to that of control, indicating that consumers would widely prefer these products. These findings could be explained by the fact that these herbs, oregano and rosemary, especially the former, are frequently used in dishes in Mediterranean cuisine, and therefore, assessors of the present study were familiar with their aroma and taste, finding these flavor notes quite acceptable. The predominance of carvacrol in ORE breads and caryophyllene (sweety, spicy, clove odor) in ROS breads, instead of 1,8-cineole (Table 5), which is one of the major components of rosemary essential oil having camphoraceous “medicinal” odor [54,55,61], seemed to lead to a higher preference of these products by assessors (Figure 7a). In general, substituting wheat flour with SWAP at a 2% level resulted in products with low overall acceptability (mean values scores < 4). Skendi et al. [9] found that the addition of dried herbs of oregano, thyme, and satureja into wheat breads at a 1% level was the maximum acceptable concentration for consumers. The SPE2% fortified product was the least pleasant, as it showed the lowest mean overall acceptability score among all tested breads (Figure 7a). Probably, carvone, which is the predominant volatile compound in spearmint, providing the distinctly fresh and pungent mentholated, minty aroma to the plant [62], was detected in SPE breads at high levels (Table 5), which did not seem to fit with an acceptable flavor of such bakery products. Similarly, Shori et al. [63] noted that as the level of spearmint aqueous extract increased in wheat bread formulations, the overall acceptability of the product by the assessors decreased.

Since the 2% SWAP-fortified bread formulations were not considered acceptable by the consumer panel, the evaluation of specific sensory attributes was conducted only for the 1%-fortified products. The distinct taste and aroma of oregano, rosemary, lemon balm, and spearmint were highly (>5 mean value scores) detectable by the assessors in breads containing the waste residues of the respective MAPs. These findings are in agreement with the instrumental analysis of volatile compounds; i.e., for the ORE-fortified breads (Table 5), high levels of carvacrol, the typical compound of oregano, were detected. Similarly, in SPE samples, the characteristic compound attributed to spearmint flavor, carvone, was also found in high amounts, while in ROS baked products, although the major volatile molecule detected was caryophyllene, some amounts of the typical components of the rosemary aroma, such as 1,8-cineol, bornyl acetate, and a-pinene, were also detected. On the other hand, citronellal, neral, and geranial, which are the major volatile compounds of lemon balm, were not detected in the breads, possibly due to the extremely low yield of lemon balm essential oil (<0.3%), and the major terpenoid found in such products was β-caryophyllene. Additionally, for the LEB breads, it appeared that terpenoids had the lowest contribution in the total volatile compounds compared to the other SWAP-fortified breads. Thus, LEB1% bread displayed the lowest mean values for its distinct aroma and taste, and ORE and ROS were the highest (Figure 7b). The latter, in combination with the high overall acceptability scores recorded for the ORE1% and ROS1% breads (Figure 7a), might indicate that oregano and rosemary aroma and taste, although they were intense for these products, are quite pleasant and acceptable to the assessors. These findings further suggest that substituting even a small amount of 1% of wheat flour with solid post-distillation residues of MAPs is sufficient to impart the characteristic taste and aroma of these plants (Figure 7b). Therefore, for all SWAP-fortified breads at 1% levels, the obtained scores for wheat aroma and taste attributes showed low intensity, and it seemed to have been replaced by the distinct aroma and taste of the respective MAPs from which the solid residues have been; i.e., all SWAP-containing products obtained high scores for ‘non-wheat aroma and taste’ perception (Figure 7c). The LEB1% bread had the mildest non-wheat aroma and taste among the SWAP-containing products. This is in agreement with the lowest score obtained by this particular product (Figure 7b) as well as the lowest levels of terpenoids among the fortified products and the predominance of 1-butanol-3-methyl, the major volatile component of control bread, in the volatile profile of LEB breads (Table 5). 

Unfortunately, a bitter taste was noted in all wheat flour-SWAP composite breads (Figure 7c), which was the reason that assessors found the products unpleasant with a 2% supplementation level (Figure 7a). However, the bitterness was not very intense at 1% SWAP and did not lead the assessors to reject the samples; among the fortified breads, the less bitter was the product with lemon balm waste. The highest score for bitter taste was given for the ROS-containing bread, probably due to the high levels of bitter diterpenes, carnosol, and carnosic acid [61] that was found in this baked product (Table 3). Moreover, the assessors identified a darker crust and crumb color in all fortified breads with the post-distillation MAPs waste residues, compared to the control sample, with the ORE1% sample presenting the darkest color for both crust and crumb (Figure 7c). Similar findings were obtained using instrumental analysis as well (Figure 1).

## 4. Conclusions

This study indicated that the incorporation of post-distillation solid waste residues of the MAPs of oregano, rosemary, lemon balm, and spearmint into wheat breads could affect some of their physicochemical properties and introduce the characteristic flavor notes of the respective plants into the bakery product. On the other hand, such fortification can enhance the content of antioxidants and result in sensorially acceptable products when the residues are used at relatively low levels of wheat flour substitution. Specifically, the addition of MAP wastes resulted in a darker crust and crumb color and a more greenish crumb color in breads fortified at both 1 and 2% levels. Instead, the incorporation of these by-products did not seem to affect the loaf specific volume, crust hardness, and crumb cohesiveness as well as crumb hardness after 4 days of storage at 25 °C, although the crumb chewiness increased, except in the case of rosemary, for both fresh and stored fortified breads. Baking decreased the concentration of most of the phenolic compounds of the added residues, compared to the theoretically expected values; however, fortification of wheat bread with the plant residues seemed to be an effective means for enhancing the content of phenolic acids, terpenoid compounds and to a lesser extent flavonoids in breads, along with an increasing antioxidant capacity of the end-products. However, solid wastes had no considerable impact on mold growth on the crumb surfaces of the fortified breads during storage, most likely due to their low fortification levels or/and the fact that they led to higher crust and crumb moisture contents in the breads than those of control. The profile of volatile compounds, determined using gas chromatography, also showed an enrichment of the fortified breads with terpenoid compounds, which were detectable by trained sensory panelists, as well as providing to the baked products the characteristic aroma of the respective plant which was pleasant to the consumers (untrained panel) only at 1% fortification level. Overall, the inclusion of 1% of solid wastes in bread formulations was sufficient to provide the aroma and taste of the respective MAPs, with the oregano waste bread obtaining the highest overall acceptability score, followed by bread with rosemary waste. This study confirms that post-distillation solid wastes of the studied MAPs are a sustainable alternative ingredient for the development of functional bakery products enriched in antioxidants that could provide beneficial effects on human health. Although the selected MAPs are native to the Mediterranean and Europe, they are common essential oil plants, while some of them have been naturalized and cultivated in several parts of the world.

In conclusion, this study gives for the first time a detailed examination of both the phenolic and aromatic profile of a SWAP-enriched bread in combination with the quality and shelf-life attributes of the baked product. These results further demonstrate that the SWAP of the selected plants represents a promising source of sustainable phenolics with high antioxidant properties. Beyond that, the present study paves the way to the valorization of SWAP of similar chemical patterns’ plants, commercialized for their essential oils all around the world.

## Figures and Tables

**Table 1:** Composition of fortified wheat breads with post-distillation solid waste residues of aromatic plants.

Sample Symbol	Wheat Flour (g)	Solid Waste of Aromatic Plant (SWAP)	Water (mL)	Baker’s Yeast (g)	Salt (g)
CON	100	-	58.5	1	2
ORE1%	99	1 g ORE	60.0	1	2
ORE2%	98	2 g ORE	61.0	1	2
ROS1%	99	1 g ROS	59.5	1	2
ROS2%	98	2 g ROS	60.5	1	2
LEB1%	99	1 g LEB	61.5	1	2
LEB2%	98	2 g LEB	62.0	1	2
SPE1%	99	1 g SPE	60.0	1	2
SPE2%	98	2 g SPE	62.0	1	2

ORE: oregano; ROS: rosemary; LEB: lemon balm; SPE: spearmint.

**Table 2:** Total phenolic content (TPC), total flavonoid content (TFC), and antioxidant activity of control bread (CON) and breads fortified with 1% and 2% post-distillation solid waste residues of oregano (ORE), rosemary (ROS), lemon balm (LEB) and spearmint (SPE), in free and total (sum of free and bound) fractions.

Fraction	Bread Sample	TPC (mg GAE/100 g Bread d.b.)	TFC (mg CATE/100 g Bread d.b.)	Antioxidant Activity (mg TE/100 g Bread d.b.)		
				ABTS	DPPH	FRAP
Free	CON	21.8 ± 1.20a	14.29 ± 2.18a	26.13 ± 0.5a	5.77 ± 0.18a	29.95 ± 0.35a
	ORE1%	59.39 ± 1.16b	23.29 ± 0.82b	70.27 ± 5.76b	37.25 ± 0.91b	75.27 ± 1.02b
	ORE2%	91.27 ± 1.43e	35.65 ± 0.47c	139.70 ± 1.64g	66.27 ± 0.09e	86.34 ± 1.56c
	ROS1%	70.8 ± 0.69c	38.20 ± 3.04c	90.29 ± 0.81d	54.42 ± 0.57d	89.43 ± 1.99c
	ROS2%	97.75 ± 2.04f	48.29 ± 0.03d	140.79 ± 3.06g	99.53 ± 2.45g	96.39 ± 0.52d
	LEB1%	78.29 ± 0.89d	53.28 ± 1.15e	109.50 ± 2.38e	76.26 ± 0.39f	119.78 ± 0.43f
	LEB2%	121.34 ± 2.49g	81.54 ± 0.20f	190.18 ± 1.96h	131.67 ± 0.78h	134.66 ± 1.81g
	SPE1%	63.17 ± 0.35b	38.96 ± 3.25c	81.89 ± 1.35c	50.18 ± 1.56c	89.35 ± 1.32c
	SPE2%	99.94 ± 3.20f	79.92 ± 2.68f	133.59 ± 0.08f	100.69 ± 0.16g	102.16 ± 0.83e
Total	CON	98.73 ± 4.71a	26.49 ± 0.91a	135.66 ± 12.93a	13.63 ± 1.14a	66.77 ± 6.11a
	ORE1%	144.63 ± 1.22b	54.18 ± 2.91b	283.83 ± 7.52c	83.21 ± 2.20b	191.27 ± 0.71c
	ORE2%	201.03 ± 2.75cd	92.78 ± 3.66d	416.63 ± 14.61e	129.78 ± 0.52d	267.34 ± 7.08d
	ROS1%	144.57 ± 2.33b	64.08 ± 1.00c	226.80 ± 12.08b	119.23 ± 2.43c	160.28 ± 10.88b
	ROS2%	202.68 ± 2.80cd	62.67 ± 2.77bc	275.44 ± 7.93bc	186.78 ± 7.36g	166.21 ± 1.07b
	LEB1%	210.84 ± 5.51d	109.00 ± 4.76e	363.75 ± 56.31d	176.05 ± 5.78f	287.81 ± 6.03e
	LEB2%	285.5 ± 6.12f	160.29 ± 2.56f	593.65 ± 25.43f	285.42 ± 8.80h	390.99 ± 0.36f
	SPE1%	197.36 ± 5.03c	54.82 ± 8.10b	275.67 ± 15.54bc	87.70 ± 3.05b	157.39 ± 0.86b
	SPE2%	252.00 ± 5.60e	103.14 ± 0.25e	342.38 ± 8.57d	150.32 ± 0.44e	200.24 ± 4.16c
	Factor	Two-Factor ANOVA—*p* values				
Free	plant	0.090	0.000	0.013	0.000	0.000
	level	0.018	0.006	0.017	0.001	0.059
	plant × level	0.991	0.371	0.537	0.684	0.675
Total	plant	0.000	0.000	0.000	0.000	0.000
	level	0.000	0.000	0.000	0.000	0.000
	plant × level	0.036	0.000	0.003	0.000	0.000

GAE: gallic acid equivalents; TE: CATE: catechin equivalents; Trolox equivalents; d.b.: dry basis. Values are means of three replicates; mean values with different letters in the same column for the same parameter and fraction are significantly different (Tukey’s test, *p* < 0.05).

**Table 3:** Major phenolic compounds (mg/g) quantified in the free extracts of dried post-distillation solid waste residues of oregano (ORE), rosemary (ROS), lemon balm (LEB), and spearmint (SPE).

Analytes	ORE	ROS	LEB	SPE
CA	0.25 ± 0.00	0.28 ± 0.06	0.28 ± 0.03	0.25 ± 0.03
RMA	17.42 ± 0.81	21.29 ± 0.47	54.10 ± 2.26	25.02 ± 2.98
SALB	4.80 ± 0.03	-	-	0.19 ± 0.02
SALA	1.11 ± 0.07	0.90 ± 0.05	0.32 ± 0.03	2.56 ± 0.09
LITHA	-	-	5.82 ± 0.46	0.24 ± 0.01
SALI	-	-	-	4.04 ± 0.13
Total PAs	23.58a	22.47a	60.52c	31.30b
CARV	26.00 ± 0.92	-	-	-
CARO	-	23.95 ± 0.17	-	-
CARA	2.48 ± 0.04	203.00 ± 1.06	1.98 ± 0.07	2.05 ± 0.07
Total PTs	28.45b	226.95c	1.98a	2.05a
VIC	2.68 ± 0.07	0.14 ± 0.01	0.07 ± 0.02	0.12 ± 0.01
API	0.04 ± 0.00	-	-	-
LUTGL	0.09 ± 0.01	0.04 ± 0.00	0.08 ± 0.02	0.93 ± 0.12
LUTRU	-	-	-	1.30 ± 0.14
NAR	0.22 ± 0.09	-	-	-
ERD	0.49 ± 0.01	-	-	-
HESP	-	0.14 ± 0.01	0.03 ± 0.00	0.85 ± 0.16
TAX	0.62 ± 0.02	-	-	-
VER	0.24 ± 0.07	0.21 ± 0.00	nd	0.74 ± 0.06
Total FLAVs	4.37c	0.87a	0.89a	3.94b
Total PCs	56.41b	250.29d	63.39c	35.25a

All analytes are expressed in mg/g dried post-distillation solid waste residue. Symbols correspond to: lower than the limit of quantification (-); caffeic acid (CA); rosmarinic acid (RMA); salvianolic acid B (SALB); salvianolic acid A (SALA); lithospermic acid A (LITHA); salvianolic acid isomer (SALI); carvacrol (CARV); carnosol (CARO); carnosic acid (CARA); vicenin-2 (VIC); apigenin (API); luteolin-7-O-glucoside (LUTGL); luteolin-7-O-rutinoside (LUTRU); naringenin (NAR); eriodyctiol (ERD); hesperidin (HESP); taxifolin (TAX); verbascoside (VER); Phenolic acids (PAs); Phenolic terpenoids (PTs); Flavonoids (FLAVs); Phenolics (PCs). Values are means of three replicates. Mean values with different letter in the same row are significantly different (Tukey’s test, *p* < 0.05).

**Table 4:** Major phenolic compounds (free and total fractions) of control bread (CON) and breads fortified with 1% and 2% post-distillation solid waste residues of oregano (ORE), rosemary (ROS), lemon balm (LEB), and spearmint (SPE).

Analytes	Fraction	CON	ORE1%	ORE2%	ROS1%	ROS2%	LEB1%	LEB2%	SPE1%	SPE2%
t-FA	F	0.27 ± 0.02	0.24 ± 0.01	0.22 ± 0.01	0.29 ± 0.01	0.28 ± 0.03	0.22 ± 0.02	0.20 ± 0.03	0.28 ± 0.04	0.25 ± 0.04
	T	7.24 ± 0.29	5.71 ± 0.11	5.77 ± 0.37	5.77 ± 0.16	5.78 ± 0.45	6.53 ± 0.4	7.35 ± 0.52	6.60 ± 0.4	7.12 ± 0.56
c-FA	F	0.23 ± 0.01	0.19 ± 0.04	0.19 ± 0.05	0.22 ± 0.02	0.20 ± 0.03	0.24 ± 0.01	0.24 ± 0.03	0.24 ± 0.06	0.24 ± 0.06
	T	2.13 ± 0.23	2.00 ± 0.13	2.41 ± 0.21	2.16 ± 0.30	2.13 ± 0.36	1.94 ± 0.01	2.05 ± 0.13	1.34 ± 0.18	1.27 ± 0.30
pCA	F	0.06 ± 0.01	0.99 ± 0.04	2.63 ± 0.04	0.03 ± 0.00	0.04 ± 0.00	1.13 ± 0.16	0.11 ± 0.00	0.06 ± 0.01	0.09 ± 0.01
	T	0.47 ± 0.08	1.46 ± 0.02	3.15 ± 0.07	0.66 ± 0.07	0.67 ± 0.09	1.79 ± 0.24	0.77 ± 0.02	0.45 ± 0.08	0.44 ± 0.08
CA	F	0.08 ± 0.01	0.39 ± 0.00	0.55 ± 0.01	0.43 ± 0.01	0.64 ± 0.02	0.49 ± 0.00	0.78 ± 0.02	0.47 ± 0.01	0.65 ± 0.02
	T	0.28 ± 0.01	1.02 ± 0.06	2.90 ± 0.26	0.78 ± 0.04	1.24 ± 0.04	6.14 ± 0.61	9.95 ± 0.44	0.71 ± 0.01	0.93 ± 0.03
RMA	F	-	15.16 ± 0.40	27.03 ± 0.24	22.46 ± 0.25	36.28 ± 0.88	58.10 ± 3.54	92.90 ± 3.87	35.32 ± 0.96	50.91 ± 0.10
SALB	F	-	1.75 ± 0.04	2.74 ± 0.16	-	-	0.31 ± 0.02	0.65 ± 0.04	0.16 ± 0.02	0.38 ± 0.01
SALA	F	-	0.76 ± 0.04	1.42 ± 0.01	0.82 ± 0.02	1.44 ± 0.02	0.25 ± 0.01	0.35 ± 0.04	2.20 ± 0.08	5.29 ± 0.21
LITHA	F	-	-	-	-	-	2.62 ± 0.05	5.24 ± 0.16	0.31 ± 0.01	0.64 ± 0.03
SALI	F	-	-	-	-	-	-	-	2.30 ± 0.34	3.54 ± 0.28
Total PAs	F	0.63a	19.47b	34.76d	24.24c	38.88d	63.35e	100.46f	41.33d	61.97e
	T	10.12a	27.84b	45.4c	32.64b	47.54c	77.66e	119.25f	49.38c	70.51d
CARV	F	-	13.50 ± 2.57	30.15 ± 2.75	-	-	-	-	-	-
CARO	F	-	-	-	41.78 ± 0.22	65.70 ± 3.15	-	-	-	-
CARA	F	-	4.22 ± 0.31	5.40 ± 0.07	111.54 ± 5.04	187.71 ± 2.92	4.61 ± 0.15	3.03 ± 0.01	2.11 ± 0.16	3.17 ± 0.05
Total PTs	F	-	17.72b	35.55c	153.32d	253.41e	4.61a	3.03a	2.11a	3.17a
VIC	F	-	3.70 ± 0.17	6.30 ± 0.03	0.20 ± 0.01	0.22 ± 0.01	0.28 ± 0.11	0.16 ± 0.01	0.21 ± 0.02	0.28 ± 0.01
API	F	-	0.14 ± 0.01	0.26 ± 0.00	-	-	-	-	-	-
LUTGL	F	-	0.10 ± 0.01	0.12 ± 0.01	0.13 ± 0.01	0.14 ± 0.01	0.17 ± 0.00	0.20 ± 0.00	0.32 ± 0.04	0.54 ± 0.01
LUTRU	F	-	-	-	-	-	-	-	3.18 ± 0.05	5.23 ± 0.05
NAR	F	-	0.32 ± 0.00	0.63 ± 0.01	-	-	-	-	-	-
ERD	F	-	0.61 ± 0.00	1.14 ± 0.05	-	-	-	-	-	-
HESP	F	-	-	-	0.51 ± 0.05	0.94 ± 0.02	0.01 ± 0.00	0.01 ± 0.00	1.78 ± 0.04	2.94 ± 0.04
TAX	F	-	0.78 ± 0.03	0.82 ± 0.06	-	-	-	-	-	-
VER	F	-	0.31± 0.01	0.33 ± 0.01	0.46 ± 0.04	0.27 ± 0.00	-	-	1.40 ± 0.14	2.64 ± 0.05
Total FLAVs	F	-	5.95d	9.59f	1.30b	1.66c	0.46a	0.37a	6.89e	11.60g
Total PCs	F	0.63a	43.14b	79.89cd	178.41f	293.95g	68.41c	103.85e	50.33b	76.78c
	T	10.12a	51.51b	90.53d	187.26f	302.61g	82.7c	122.64e	58.38b	85.41c

All analytes are expressed in mg/100 g bread (dry basis). Symbols correspond to: lower than the limit of quantification (-); trans-ferulic acid (t-FA); cis-ferulic acid (c-FA); p-coumaric acid (pCA); caffeic acid (CA); rosmarinic acid (RMA); salvianolic acid B (SALB); salvianolic acid A (SALA); lithospermic acid A (LITHA); salvianolic acid isomer (SALI); carvacrol (CARV); carnosol (CARO); carnosic acid (CARA); vicenin-2 (VIC); apigenin (API); luteolin-7-O-glucoside (LUTGL); luteolin-7-O-rutinoside (LUTRU); naringenin (NAR); eriodyctiol (ERD); hesperidin (HESP); taxifolin (TAX); verbascoside (VER); Phenolic acids (PAs); Phenolic terpenoids (PTs); Flavonoids (FLAVs); Phenolic compounds (PCs); F, free phenolic extract; T, total phenolic extract (sum of free and bound). Values are means of three replicates. Mean values with different letter in the same row are significantly different (Tukey’s test, *p* < 0.05).

**Table 5:** Relative concentration (area %) of the most abundant volatile compounds detected using SPME/GC/MS chromatography in post-distillation solid waste residues of oregano (ORE), rosemary (ROS), lemon balm (LEB), and spearmint (SPE) as well as control bread (CON) and breads fortified with 1% and 2% of the above solid wastes.

Volatile Compound	Distilled Solid Wastes				Breads								
	ORE	ROS	LEB	SPE	CON	ORE1%	ORE2%	ROS1%	ROS2%	LEB1%	LEB2%	SPE1%	SPE2%
*Aldehydes*													
butanal-2-methyl	-	-	-	-	0.54	0.23	0.25	0.77	0.45	0.66	0.72	0.51	0.28
pentanal	-	-	0.63	-	0.76	-	-	-	-	0.99	1.05	0.71	0.36
hexanal	-	-	-	-	7.70	0.75	0.21	6.55	4.11	9.42	7.55	4.66	2.48
heptanal	-	-	-	-	4.06	0.27	-	2.82	1.61	3.27	3.03	1.88	1.01
2-heptenal	-	-	-	-	-	0.22	0.36	3.59	1.75	12.39	8.17	2.62	3.60
octanal	-	-	-	-	1.23	-	-	-	-	-	-	-	-
2-hexenal	-	-	-	-	-	-	-	0.97	0.64	1.66	1.56	0.46	0.57
benzaldehyde	-	-	-	-	2.64	0.53	0.16	1.68	1.19	-	-	0.81	0.45
nonanal	-	-	-	-	1.75	-	-	-	0.50	-	-	-	-
*Alcohols*													
1-butanol-3-methyl	-	-	-	-	26.96	1.97	0.58	20.38	9.95	28.32	26.67	10.63	6.24
1-butanol-2-methyl	-	-	-	-	9.07	0.97	0.30	9.35	4.84	11.28	10.86	4.78	2.66
amylol	-	-	-	-	1.42	-	-	-	-	-	-	0.62	0.44
hexanol	-	-	-	-	10.32	-	-	6.24	2.84	8.06	7.38	3.07	2.10
heptanol	-	-	-	-	1.28	-	-	-	-	-	-	-	-
1-octen-3-ol	-	-	-	-	1.19	-	0.16	1.21	0.77	2.80	3.19	0.93	1.39
1-dodecanol	-	-	-	-	-	0.27	0.04	1.50	1.25	1.63	1.85	-	-
furfuryl alcohol	-	-	-	-	2.32	-	-	-	-	-	-	-	-
phenethyl alcohol	-	-	-	-	5.50	-	-	-	-	-	-	-	-
*Ketones*													
2-heptanone	-	-	-	-	1.20	-	-	-	-	-	-	0.71	0.47
3-heptanone	-	-	-	-	-	-	-	-	-	3.30	3.55	1.11	1.59
*Furans*													
2-amylfuran	-	-	-	-	3.91	0.28	0.15	1.53	1.20	1.19	2.19	0.88	0.49
*Esters*													
propyl phenylacetate	-	-	-	-	1.32	0.26	0.07	1.20	0.79	1.67	1.59	1.27	0.75
1-hexyl acetate	-	-	-	-	-	-	-	-	2.94	1.30	-	-	-
*Terpenoids*													
α-pinene	-	1.56	-	-	-	-	-	2.38	2.67	-	-	-	-
β-pinene	-	1.40	0.65	0.28	-	-	-	1.72	1.93	-	-	-	-
β-myrcene	-	1.16	0.44	-	-	-	-	-	-	-	-	-	-
α-terpinene	-	-	-	-	-	-	-	-	1.03	-	-	-	-
p-cymene	-	2.66	-	0.23	-	0.65	0.27	1.05	1.48	-	-	-	-
limonene	-	2.03	0.83	-	-	-	-	0.55	0.90	-	-	-	-
β-phellandrene	-	-	0.38	-	-	-	-	-	-	-	-	-	-
1,8-cineole	-	16.98	-	4.62	-	-	-	4.63	4.34	-	-	-	-
γ-terpinene	-	-	0.20	-	-	-	-	-	-	-	-	-	-
linalool	-	1.77	0.18	9.93	-	-	-	-	-	-	-	-	-
citronellal	-	-	0.50										
borneol	-	3.77	-	0.78	-	-	-	-	-	-	-	-	-
terpinen-4-ol	0.52	1.41	-	1.08	-	-	-	-	-	-	-	-	-
α-terpineol	-	5.54	-	1.08	-	-	-	1.07	2.17	-	-	-	-
neral	-	-	2.76										
carvone	-	-	-	35.14	-	-	-	-	-	-	-	54.80	58.88
geraniol	-	-	0.80	-	-	-	-	-	-	-	-	-	-
bornyl acetate	-	6.04	-	1.18	-	-	-	4.81	6.88	-	-	-	-
geranial	-	-	5.50	-	-	-	-	-	-	-	-	-	-
thymoquinone	-	-	-	-	-	46.08	45.29	-	-	-	-	-	-
thymol	8.37	-	-	-	-	-	-		-	-	-	-	-
carvacrol	77.78	-	-	-	-	43.82	49.47	-	-	-	-	-	-
α-copaene	-	-	4.36	-	-	-	-	-	-	-	-	-	-
β- bourbonene	-	-	1.63	5.07	-	-	-	-	-	-	-	6.26	8.21
α-cubebene	-	-	0.62	-	-	-	-	-	-	-	-	-	-
β-elemene	-	-	1.70	-	-	-	-	-	-	-	-	-	-
β-caryophyllene	3.05	37.51	39.00	3.34	-	1.50	1.16	23.48	40.97	6.38	13.57	0.64	0.69
β-copaene	-	-	0.58	-	-	-	-	-	-	-	-	-	-
α-bergamotene	-	-	1.10	5.07	-	-	-	-	-	-	-	-	-
aromadendrene	-	-	-	1.07	-	-	-	-	-	-	-	-	-
α-humulene	0.35	2.51	3.86	-	-	-	-	-	-	-	-	-	-
β-farnesene	-	-	-	2.22	-	-	-	-	-	-	-	-	-
cis-muurola-4(15),5-diene	-	-	-	2.00	-	-	-	-	-	-	-	0.78	0.54
γ-muurolene	-	-	1.40	-	-	-	-	-	-	-	-	-	-
germacrene D	-	-	20.60	6.07	-	-	-	-	-	1.12	3.40	0.89	1.94
α-muurolene	-	-	1.70	-	-	-	-	-	-	-	-	-	-
γ-cadinene	-	-	1.53	2.07	-	-	-	-	-	-	-	-	-
δ-cadinene	-	-	5.80	-	-	-	-	-	-	-	-	-	-
α-cadinene	-	-	1.50	-	-	-	-	-	-	-	-	-	-
caryophyllene oxide	1.96	3.71	1.40	-	-	0.41	0.41	0.88	1.90	-	-	-	-

Compounds with (-) was below the limit of detection. Values are means of three replicates.

## Data Availability

Data is contained within the article or Supplementary Material.

## Supplementary Materials

The following supporting information can be downloaded at: https://www.mdpi.com/article/10.3390/foods12214007/s1, Figure S1: Representative HPLC chromatograph showing the main phenolic components of the control (a) and fortified breads with 2% ORE (b), 2% ROS (c), 2% LEB (d) and 2% SPE (e) in the free extracts; inset graph shows the respective chromatogram at 320 nm of control bread in the bound extract (f). Sample symbols correspond to: ORE, oregano; ROS, rosemary; LEB, lemon balm; SPE, spearmint. Peaks correspond to: VIC, vicenin-2; CA, caffeic acid; LUTGL, luteolin-7-O-glucoside; LUTRU, luteolin-7-O-rutinoside; pCA, p-coumaric acid; VER, verbascoside; TAX, taxifolin; t-FA, trans-ferulic acid; c-FA, cis-ferulic acid; HESP, hesperidin; RMA, rosmarinic acid; SALB, salvianolic acid B; SALA, salvianolic acid A; IS, internal standard; LITHA, lithospermic acid A; SALI, salvianolic acid isomer; ERD, eriodictyol; NAR, naringenin; API, apigenin; CAR, carvacrol; CARO, carnasol; CARA, carnosic acid; ni, not identified.
