# Supplementary material for: Physicochemical Characteristics, Antioxidant Properties, Aroma Profile, and Sensory Qualities of Value-Added Wheat Breads Fortified with Post-Distillation Solid Wastes of Aromatic Plants
Source: Foods. 2023 Nov 2;12(21):4007. doi: 10.3390/foods12214007 (PMC10648853; doi:10.3390/foods12214007)
Supplement: Supplementary file 1 [file foods-12-04007-s001.zip › foods-2672834-supplementary.pdf]

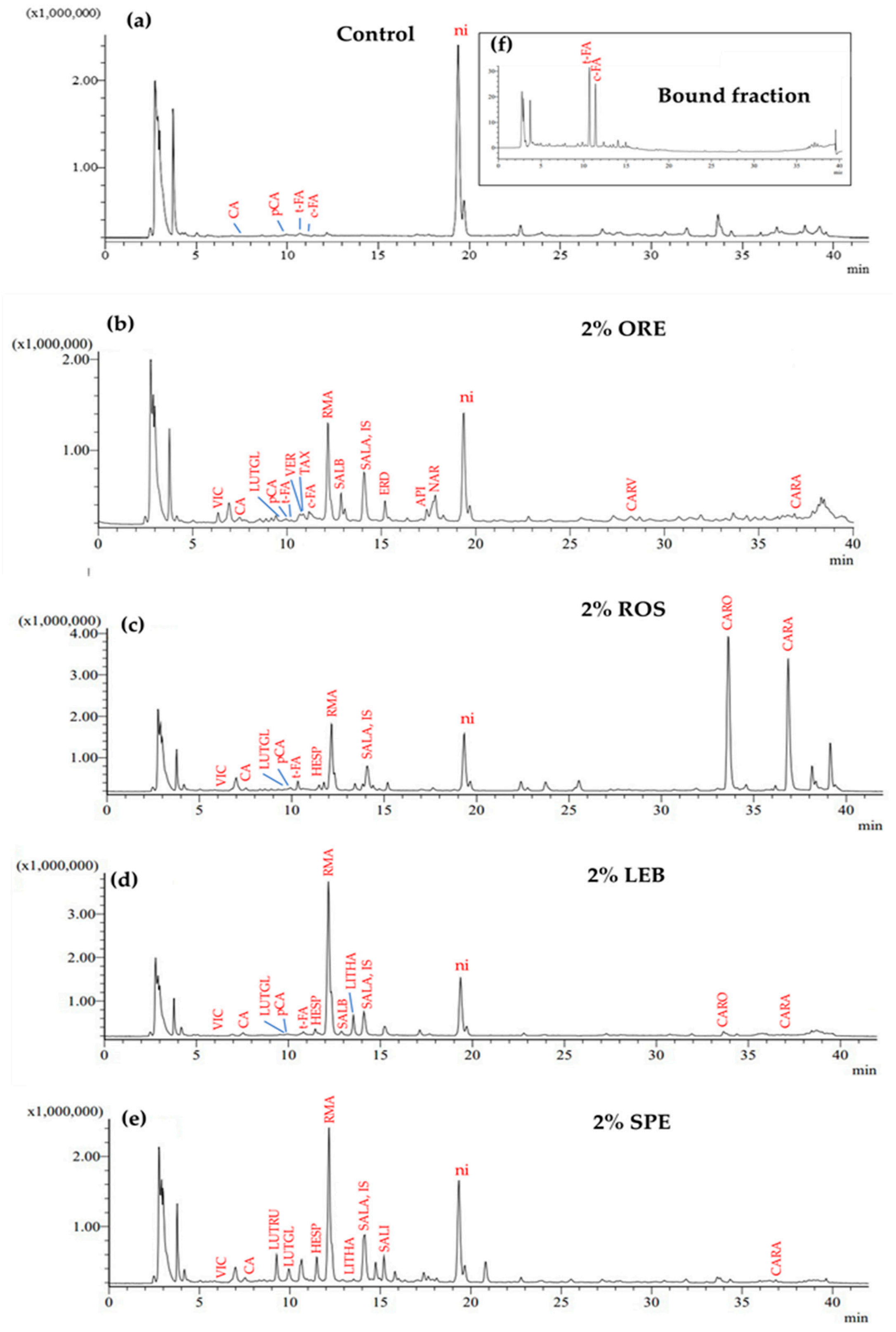

Figure S1. Representative HPLC chromatographs showing the main phenolic components in the free extracts of the control (a) and fortified breads with 2% ORE (b), 2% ROS (c), 2% LEB (d) and 2% SPE (e); inset graph shows the respective chromatogram at 320 nm of control bread for the bound extract (f). Sample symbols correspond to: ORE, oregano; ROS, rosemary; LEB, lemon balm; SPE, spearmint. Peaks correspond to: VIC, vicenin-2; CA, caffeic acid; LUTGL, luteolin-7-O-glucoside; LUTRU, luteolin-7-O-rutinoside; pCA, p-coumaric acid; VER, verbascoside; TAX, taxifolin; t-FA, trans- ferulic acid; c-FA, cis-ferulic acid; HESP, hesperidin; RMA, rosmarinic acid; SALB, salvianolic acid B; SALA, salvianolic acid A; IS, internal standard; LITHA, lithospermic acid A; SALI, salvianolic acid isomer; ERD, eriodictyol; NAR, naringenin; API, apigenin; CAR, carvacrol; CARO, carnasol; CARA, carnosic acid; ni, not identified.
